# miR-146b-5p mediates p16-dependent repression of IL-6 and suppresses paracrine procarcinogenic effects of breast stromal fibroblasts

**DOI:** 10.18632/oncotarget.4933

**Published:** 2015-08-17

**Authors:** Mysoon M. Al-Ansari, Abdelilah Aboussekhra

**Affiliations:** ^1^ Department of Molecular Oncology, King Faisal Specialist Hospital and Research Center, Riyadh, Saudi Arabia; ^2^ Department of Microbiology, Faculty of Science and Medical Studies, King Saud University, Riyadh, Saudi Arabia

**Keywords:** p16^INK4A^, IL-6, miR-146b-5p, breast cancer, cancer-associated fibroblasts

## Abstract

Increasing evidence support the critical roles of active stromal fibroblasts in breast cancer development and spread. However, the mediators and the mechanisms of regulation are still not well defined. We have shown here that the tumor suppressor p16^INK4A^ protein inhibits the pro-carcinogenic effects of breast stromal fibroblasts through repressing the expression/secretion of IL-6. Indeed, p16^INK4A^ suppresses IL-6 at the mRNA and protein levels. This effect is mediated trough miR-146b-5p, which inhibits IL-6 expression through a specific sequence at the IL-6 3′UTR. In addition, we present clear evidence that miR-146b-5p inhibition is sufficient to transactivate breast stromal fibroblasts, which promote epithelial-to-mesenchymal-transition in breast cancer cells in a paracrine manner. By contrast, ectopic expression of miR-146b-5p in active fibroblasts abrogated their pro-carcinogenic effects. The physiological importance of miR-146b-5p inhibition was revealed by showing that the levels of pre-miR-146b-5p as well as its mature form are reduced in cancer-associated fibroblasts as compared with their normal adjacent counterparts from cancer-free tissues isolated from the same patients. Interestingly, treatment of active breast stromal fibroblasts with curcumin increased the level of the p16^INK4A^ coding *CDKN2A* mRNA and miR-146b-5p and suppressed IL-6, which confirms the repressive effect of these two tumor suppressor molecules on IL-6, and shows the possible “normalization” of cancer-related active fibroblasts. These results show that miR-146b-5p has non-cell-autonomous tumor suppressor function through inhibition of IL-6, suggesting that targeting this microRNA in breast stromal fibroblasts could be of great therapeutic value.

## INTRODUCTION

In a tumor, cancer cells are surrounded by various types of cells, which compose the tumor microenvironment. Stromal fibroblasts, which include normal fibroblasts and myofibroblasts, are considered sustaining players in the tumor microenvironment. In fact, active cancer-associated fibroblasts (CAFs) support the growth and dissemination of cancer cells in a non-cell-autonomous manner through secretion of various chemokines, cytokines and growth factors [1, 2]. These secretions are under the control of several tumor suppressor genes, including the cyclin-dependent kinase inhibitor p16^INK4A^ (p16) [3, 4]. Indeed, we have recently shown the role of p16 downregulation in the activation of breast stromal fibroblasts [3, 5]. This effect is mediated through the up-regulation and increase in the secretion of SDF1 [3]. These findings unraveled non-cell-autonomous tumor suppressor functions of p16 through repressing the paracrine pro-carcinogenic effects of breast stromal fibroblasts.

In addition to SDF1, interleukin-6 (IL-6) is also an important secreted soluble factor that plays crucial role in the crosstalk between cancer cells and their microenvironment. IL-6 is a multifunctional cytokine implicated in both innate and acquired immune responses, hematopoiesis, inflammation as well as in the regulation of growth and differentiation of cancer cells [6]. Breast cancer tissues express high levels of IL-6 as compared with matched normal tissues and these levels increase with tumor grade [7]. We have recently shown that IL-6 is involved in breast cancer cells–dependent activation of breast stromal fibroblasts [5]. Moreover, it has been recently shown that CAFs from human breast and ovarian tumors express high levels of IL-6 [8, 9]. The IL-6 gene is tightly regulated at both the transcriptional and post-transcriptional levels [10, 11]. Recently, several microRNAs (miRNAs) were shown to be involved in this regulation [12].

miRNAs are defined as single stranded short (19–25 nucleotides in length), noncoding endogenous RNAs, which negatively regulate plethora of genes implicated in various biochemical pathways. Therefore, alteration of their expression is related to various diseases including cancer [13, 14]. miRNAs act as tumour suppressors or oncogenes, and are involved in various steps of the carcinogenesis process, including metastasis [15]. miR-146b-5p is an important tumour suppressor miRNA [16]. Indeed, miR-146b-5p was shown to suppress EGFR expression and reduces the migration and invasion of glioma and breast cancer cells *in vitro* [17–19], as well as breast cancer metastasis [20]. Furthermore, miR-146b-5p inhibits NF-kB activity and the inflammatory pathway in breast cancer cells [17, 21].

In the present study, we have shown that the tumor suppressor p16 protein suppresses the expression of IL-6 in a miR-146b-5p-dependent manner. We present also clear evidence that miR-146b-5p has non-cell-autonomous tumor suppressive functions.

## RESULTS

### p16 represses IL-6 expression

**Figure 1:**
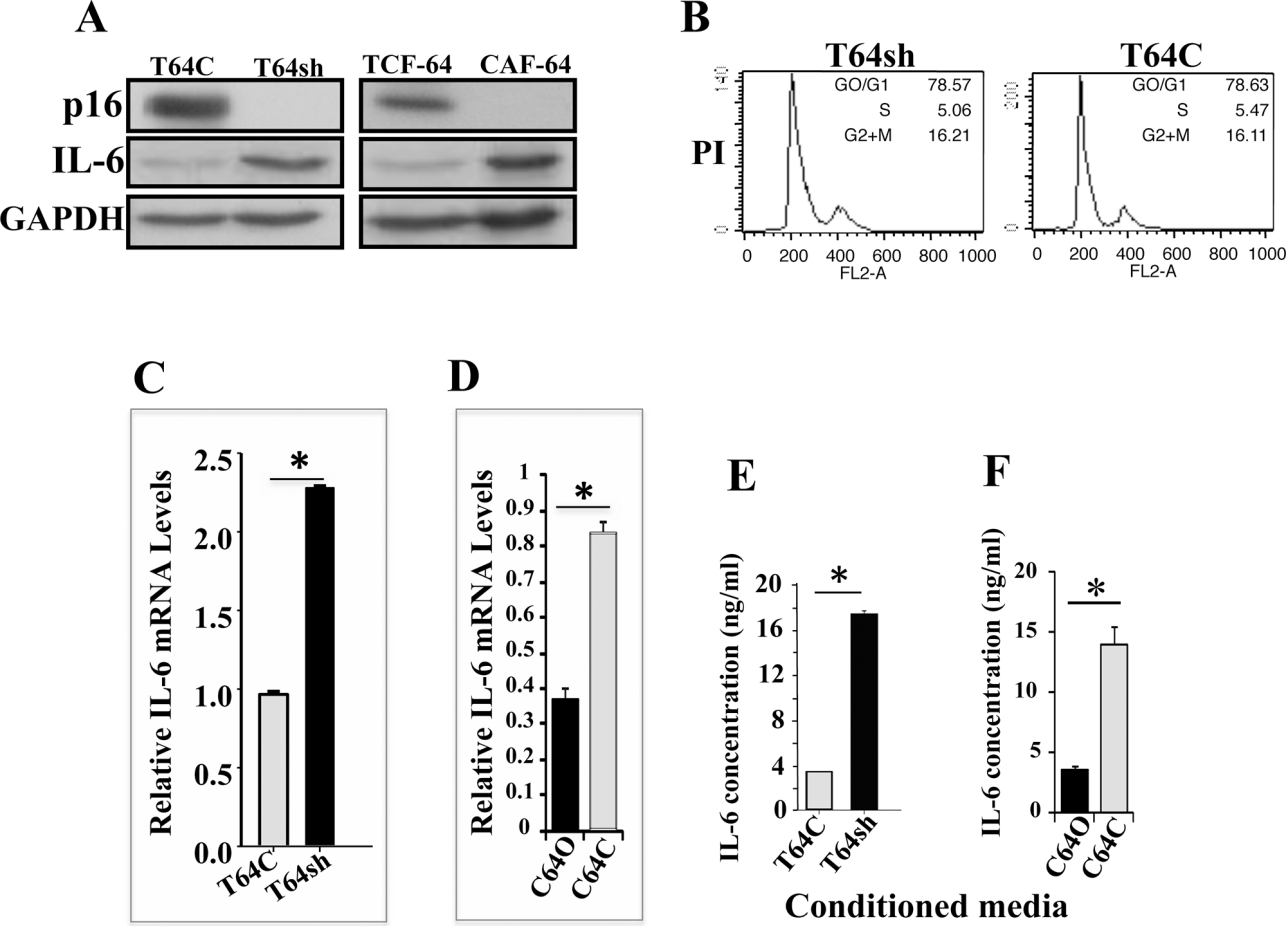
p16 suppresses IL-6 expression and secretion **A.** Whole cell lysates were prepared from the indicated cells and 60 μg of proteins were utilized for immunoblotting analysis using antibodies against the indicated proteins. **B.** DNA content of exponentially growing cells was determined by flow cytometry upon PI staining. **C.** and **D.** Total RNA was extracted from the indicated cells, and the amount of the *IL-6* mRNA was assessed by qRT-PCR. Error bars represent means ± S.D. *p value < 0.000008. **E.** and **F.** Conditioned media from the indicated cells were collected after 24 h and the levels of the indicated proteins were determined by ELISA and were presented in the respective histograms. Error bars represent means ± S.D. *p value < 0.00025.

We have recently shown that p16 suppresses the pro-carcinogenic effects of breast stromal fibroblasts [3]. Since these cells secrete IL-6, which is a major player in breast carcinogenesis, especially the carcinoma-stroma reciprocal interplay, we sought to investigate the possible role of p16 in repressing the expression of IL-6. Therefore, we first assessed the levels of p16 and IL-6 in CAF-64 cells and their counterparts isolated from histologically normal part of the same breast (TCF-64) by immunoblotting using specific antibodies, and GAPDH was utilized as internal control. Figure 1A shows an inverse correlation between the expression of p16 and IL-6. Indeed, while p16 was undetectable in CAF-64 cells, IL-6 was highly expressed in these cells as compared to their control TCF-64 cells (Figure 1A). To confirm this link between p16 and IL-6, we knocked-down p16 in TCF-64 cells using specific shRNA (T64sh), while a scrambled sequence was used as control (T64C) [3], and then we assessed the levels of both proteins. Figure 1A shows that p16 down-regulation led to strong increase in the IL-6 level, which mirrors their expression in the CAF/TCF-64 pair. To rule out the possible implication of the cell cycle in this p16-related effect, we studied the cell cycle distribution of T64sh and T64C exponentially growing cells by flow cytometry. Figure 1B shows similar number of cells in the various phases of the cell cycle in both cell cultures, indicating that p16 down-regulation did not affect the cell cycle distribution, which excludes the possible implication of cell cycle in p16-dependent repression of IL-6 expression.

Next, we investigated the effect of p16 on the IL-6 mRNA. Therefore, total RNA was extracted from T64C and T64sh cells, and quantitative reverse transcription-polymerase chain reaction (qRT-PCR) using specific primers was performed. Figure 1C shows that the mRNA level of IL-6 doubled in T64sh cells as compared to the control T64C cells, indicating that the IL-6 mRNA level is regulated in a p16-dependent manner. To further verify this, we ectopically expressed the p16 coding *CDKN2A* gene in the p16-deficient CAF-64 cells (C64O), while an empty vector was used as control (C64C) [3]. Total RNA was purified and amplified by qRT-PCR. Figure 1D shows 2.4 fold decrease in the level of the IL-6 mRNA in C64O cells as compared to C64C cells. This shows that p16 represses the IL-6 mRNA expression in breast stromal fibroblasts.

Additionally, we studied the effect of p16 on the secretion of the IL-6 protein. To this end, T64C and T64sh cells were cultured in serum-free media (SFM) for 24 h, and the resulting serum-free conditioned media (SFCM) were collected and the level of secreted IL-6 was assessed by ELISA. Figure 1E shows that p16 knockdown increased the secretion of the IL-6 protein (4.5 fold) as compared to the control cells. On the other hand, ectopic expression of the *CDKN2A* gene significantly reduced the secreted level of the IL-6 protein (3 fold) as compared to the control cells (Figure 1F). This indicates that p16 suppresses the secretion of the IL-6 protein from breast stromal fibroblasts.

### The procarcinogenic effects of p16-deficient stromal fibroblasts are IL-6-dependent

**Figure 2:**
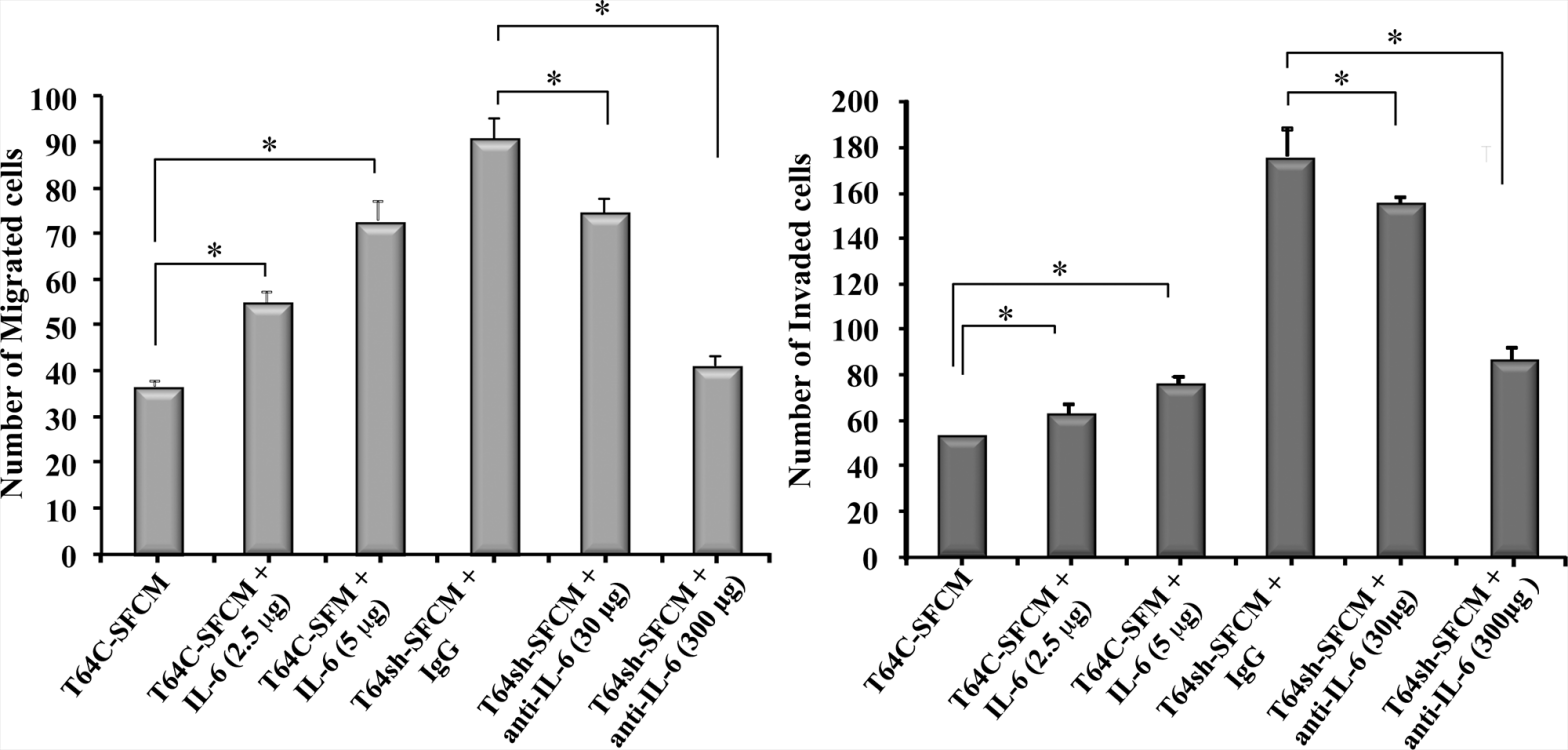
The pro-migratory/invasiveness effects of p16-deficient fibroblasts are IL-6-dependent MDA-MB-231 cells were seeded onto the upper compartment of the migration and invasion plates, and then were incubated for 24 h in the presence of SFCM from the indicated cells. Different concentrations of IL-6 or its inhibitory antibody were added as shown. The numbers of migrated and invaded cells were represented in histograms. Error bars represent means ± S.D. *p value < 0.007.

We have previously shown that p16 down-regulation in breast stromal fibroblasts activates these cells and enhances their paracrine pro-invasive/migratory effects [3]. To ascertain whether these effects are IL-6-related, we first inhibited IL-6 in SFCM from T64sh cells (T64sh-SFCM) using 2 concentrations of specific inhibitory antibody (30 and 300 ng/ml) for 24 h. SFCM from control cells (T64C-SFCM) and IgG antibody added to T64sh-SFCM were utilized as controls. These media were used to study the effect on the invasion/migration abilities of MDA-MB-231 breast cancer cells using Boyden chambers, either matrigel coated (invasion) or non-coated (migration). Figure 2 shows that the inhibition of IL-6 in T64sh-SFCM significantly reduced the migration/invasion abilities of MDA-MB-231 cells in a concentration-dependent manner. This shows that IL-6 inhibition inhibits the paracrine pro-invasive/migratory effects of p16 down-regulation in breast stromal fibroblasts. To confirm this effect, pure IL-6 protein (2.5 and 5 μg/ml) was added to SFM, and then was used to assess the effect on the migration/invasion abilities of MDA-MB-231 cells as described above. Figure 2 shows an IL-6-dependent increase in the migration/invasion capacities of breast cancer cells as compared to the negative control. These results indicate that IL-6 is a potent mediator of the paracrine pro-migratory/invasiveness capacities of p16 deficient breast stromal fibroblasts.

### p16 inhibits IL-6 expression in a miR-146b-5p-dependent manner

**Figure 3:**
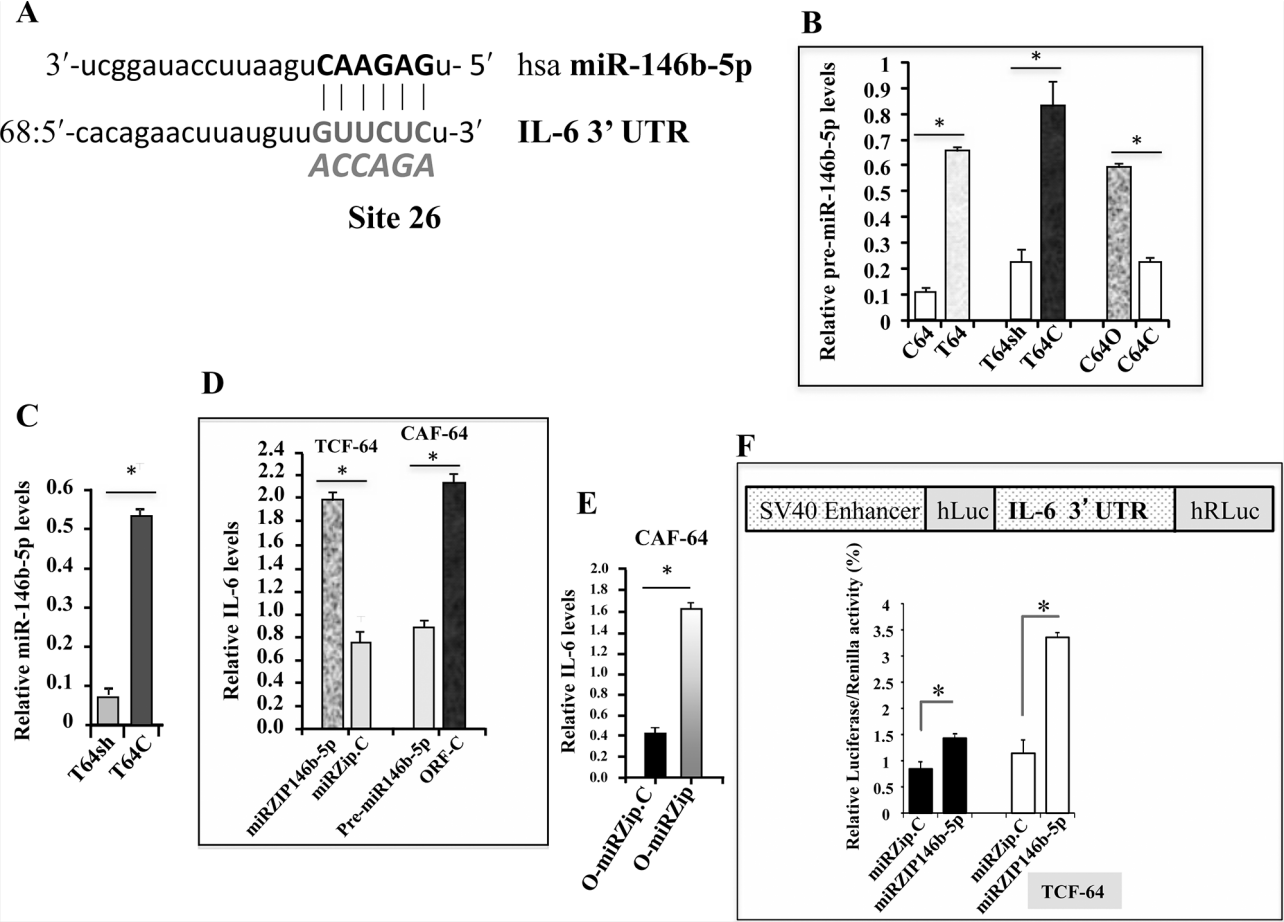
miR-146b-5p suppresses IL-6 **A.** Sequence alignment of human miR-146b-5p binding site in the *IL-6* 3′UTR, showing also the mutated sequence (bolded italic) **B, C, D.** and **E.** Total RNA was extracted from the indicated cells, and the levels of pre-miR-146b-5p, mature miR-146b-5p and *IL-6* were assessed by qRT-PCR. Error bars represent means ± S.D. *p value < 0.0009. **F.** Upper panel, schematic representation of the luciferase reporter vector bearing the miR-146b-5p binding site in the *IL-6* 3′UTR, Lower panel, cells were stably transfected with the luciferase reporter vector bearing either the wild type *IL-6* 3′UTR (black bars) or a mutated sequence (shown in a) for the binding site of miR-146b-5p (white bars). The reporter activity was assessed at 48 h post-transfection. Data (Mean ±SEM, *n* = 3) were presented as % change in reporter activity as compared to the negative control cells. *p value < 0.0078.

We have recently shown that p16, in association with Sp1 and CDK4, positively controls the expression of miR-146b-5p [16]. Accordingly, we speculated that miR-146b-5p might act as a downstream effector of p16 in suppressing IL-6. Therefore, we first made use of miRNA data bases to search for potential miR-146b-5p binding sites in the 3′ UTR of the *IL-6* transcript. We have found an important site with high complementarity located at site 68 with a miR SVR score of −0.1042 (Figure 3A). Subsequently, we have tested the affect of p16 on the expression of miR-146b-5p. Total RNA was prepared from CAF-64, TCF-64, T64sh, T64C, C64O and C64C cells, and the level of pre-miR-146b-5p was assessed by qRT-PCR. The pre-miR-146b-5p level was 5.5 fold lower in CAF-64 as compared to its corresponding TCF-64 cells (Figure 3B). Similarly, the pre-miR-146b-5p level was markedly decreased (3.5 fold) in T64sh as compared to T64C cells (Figure 3B). However, the pre-miR-146b-5p level was 3 fold higher in C64O cells as compared to their relative control C64C (Figure 3B). Similar effect was observed on the mature form of miR-146b-5p (Figure 3C). This indicates that p16 induces miR-146b-5p in breast stromal fibroblasts.

We have next investigated the effect of miR-146b-5p on the expression of IL-6. Therefore, total RNA was purified from TCF-64 cells expressing miRZip-146b-5p (miRZip), an anti-miR146b-5p inhibitor, and miRZip-146b-5p-control (miRZip-C), and qRT-PCR was performed to assess the IL-6 mRNA level. Figure 3D shows that miR-146b-5p inhibition increased 2.5 fold the IL-6 mRNA level in miRZip fibroblasts as compared to the control cells. To confirm miR-146b-5p-dependent modulation of IL-6 expression, we infected p16-defective CAF-64 cells, which express high level of IL-6, with either pre-miR-146b-5p (pre-miR) or a control lentivirus-based vector (pre-miR-C). Total RNA was prepared and the IL-6 mRNA level was assessed by qRT-PCR. Interestingly, ectopic expression of pre-miR-146b-5p significantly repressed IL-6 as compared to control cells (Figure 3D). These results indicate that miR-146b-5p represses IL-6 expression in breast stromal fibroblasts.

To show the role of miR-146b-5p in mediating the p16-related inhibition of IL-6 expression, we made use of CAF-64 cells that ectopically express p16 (C64O), and then miR-146b-5p was inhibited using miRZip-146b-5p while miRZip-146b-5p-control was used for comparison. Figure 3E shows increase in the IL-6 mRNA level in C64O cells wherein miR-146b-5p was inhibited as compared to control cells. This indicates that the p16-dependent inhibition of IL-6 is mediated through miR-146b-5p.

To further confirm the role of miR-146b-5p in repressing the expression of IL-6, we investigated the potential contribution of the miR-146b-5p binding site in the *IL-6* mRNA 3′UTR on the regulation of IL-6 expression. To this end, wild-type *IL-6* 3′UTR or the mutated sequence for the miR-146b-5p binding site were inserted into a luciferase/Renilla reporter vector (Figure 3F, upper panel) and were introduced into miRZip and their corresponding control cells miRZip-C. The reporter activity fused to the intact sequence of the *IL-6* 3′UTR was significantly induced in miRZip cells as compared to the control cells (Figure 3F, lower panel). Similarly, the activity increased also when mutated miR-146b-5p binding site was used in control cells (Figure 3F, lower panel). Interestingly, the activity was further higher by mutating the putative miR-146b-5p binding site within the 3′UTR of the *IL-6* mRNA in miRZip cells (Figure 3F, lower panel). This demonstrates that the miR-146b-5p-dependent repression of IL-6 is mediated through binding to its seeding sequence in the *IL-6* 3′UTR.

### miR-146b-5p inhibits the procarcinogenic effects of breast stromal fibroblasts

**Figure 4:**
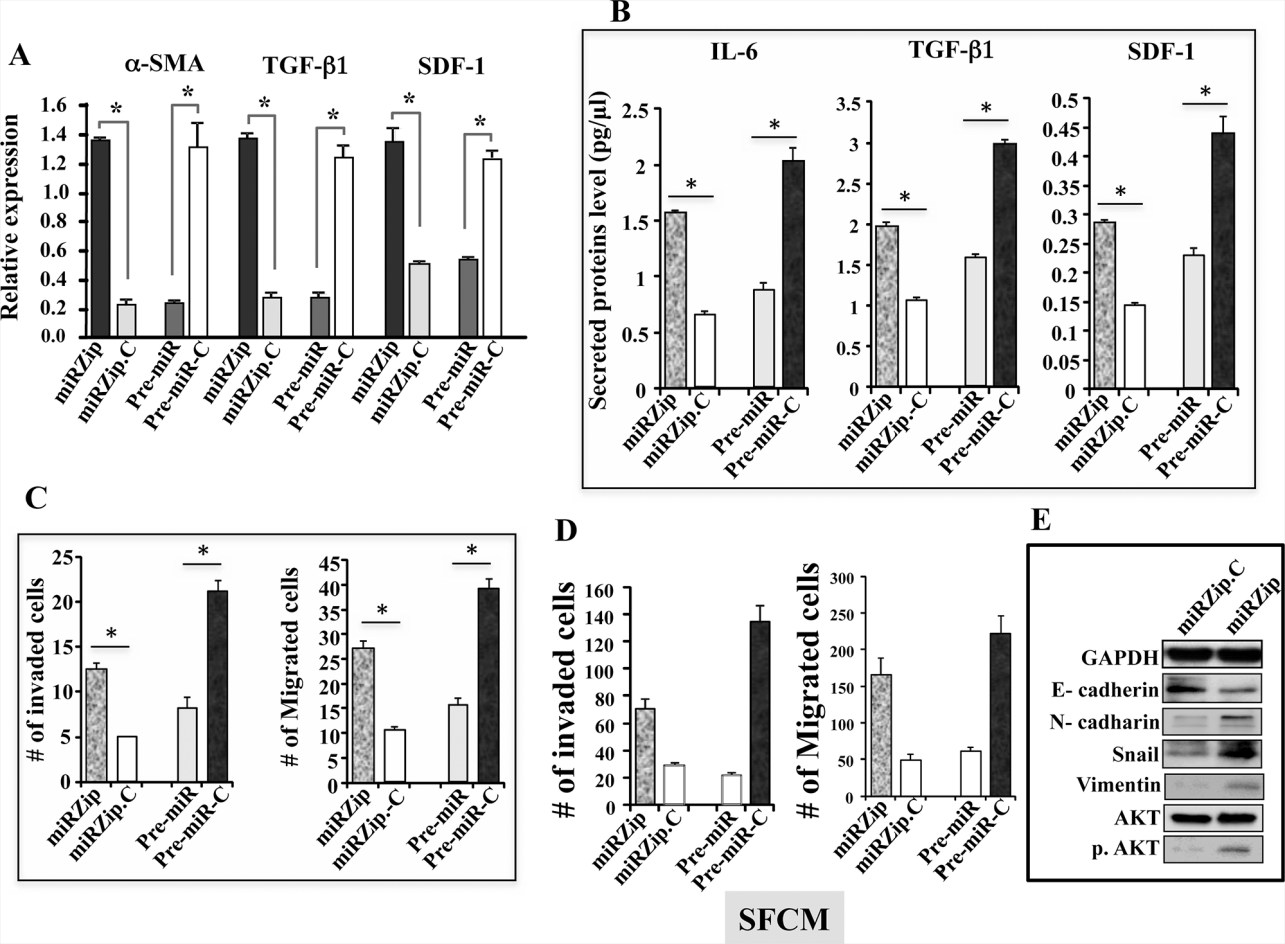
miR-146b-5p represses breast stromal fibroblasts **A.** Total RNA was extracted from the indicated cells, and the mRNA levels of the indicated genes were assessed by qRT-PCR. Error bars represent means ± S.D. *p value < 0.012. **B.** Conditioned media from the indicated cells were collected after 24 h, and then the secreted levels of the indicated proteins were determined by ELISA and were presented in the respective histograms. Error bars represent means ± S.D. *p value < 0.02. **C.** Cells (4 × 10^5^) were cultured on the upper compartments of BioCoat matrigel chambers in the presence of SFM. After 24 h of incubation, cells were stained with Diff-Quick stain then counted, and the average numbers of invasive and migrated cells are depicted in the histograms, error bars represent means ± S.D. *p value < 0.025. **D.** SFCM were collected after 24 h of incubation from the indicated cells, and were added independently into the lower compartments of 24-well BD BioCoat plates. 10^5^ MDA-MB-231 cells were seeded onto the upper compartments of the migration and invasion plates and incubated for 24 h in the presence of SFM. The number of migrated/invaded cells was represented in histograms. Error bars represent means ± S.D. **E.** Whole cell lysates were prepared from MDA-MB-231 cells that were treated for 24 h with SFM (used as control) or SFCM collected from the indicated cells. Immunoblotting analysis was performed using antibodies against the indicated proteins.

Since low p16 and high IL-6 levels are related to active fibroblasts, we sought to study the role of miR-146b-5p on the transactivation of breast stromal fibroblasts. To this end, the previously prepared total RNA from miRZip, miRZip-C, pre-miR and pre-miR-C cells was used to assess the level of the α-SMA, SDF-1 and TGF-β 1 mRNAs (3 major markers of myofibroblasts) by qRT-PCR. Figure 4A shows that miR-146b-5p inhibition increased the mRNA levels of α-SMA, TGF-β1 and SDF-1 (6.5, 4 and 2.5 fold, respectively) as compared to the control cells. Next, pre-miR-146–5p was ectopically expressed in CAF-64 cells, and then the α-SMA, TGF-β 1 and SDF-1 mRNA levels were examined. The relative levels of these mRNAs were strongly decreased (6.5, 4.5 and 2.25 fold, respectively) in pre-miR-146b-5p expressing cells as compared to the relative control cells (Figure 4A). This indicates that miR-146b-5p plays an important role in suppressing the expression of these markers of active fibroblasts.

Next, the secreted levels of IL-6, TGF-β 1 and SDF-1 were assessed by ELISA from SFCM collected from the same cells described above. Figure 4B shows that the secreted levels of these proteins were modulated in a miR-146b-5p-dependent manner. Indeed, the secreted levels of these proteins increased significantly in cells where miR-146b-5p was inhibited, while they were reduced in cells expressing pre-miR146–5p (Figure 4B). This indicates that miR-146b-5p suppresses the secretion of IL-6, TGF-β1 and SDF-1 in breast stromal fibroblasts.

To further confirm the role of miR-146b-5p inhibition in the activation of breast stromal fibroblasts, we investigated the effect of this miRNA on the invasion/migration of these cells. To this end, miRZip/miRZip-C as well as pre-miR/pre-miRC fibroblast cells cultured in SFM were added to the upper wells of the Boyden chambers either matrigel-coated (invasion) or uncoated (migration), and complete media (CpM) was placed in the lower chambers of the inserts and used as chemoattractant. Cells were incubated for 18 h, stained with Diff-Quick stain, and then were counted. Figure 4C shows that the invasion and migration abilities of miRZip cells were significantly higher than the abilities of the corresponding control cells. By contrast, the ectopic expression of pre-miR-146b-5p reduced by 2.5 fold the invasiveness and the migratory capabilities as compared to the control cells (Figure 4C). These results indicate that miR-146b-5p, like p16, suppresses both the migratory and the invasiveness abilities of breast stromal fibroblasts.

### miR-146b-5p-defective stromal fibroblasts promote epithelial–to-mesenchymal transition in breast cancer cells

Next, we investigated the effect of miR-146b-5p down-regulation or up-regulation in breast stromal fibroblasts on the invasion/migration abilities of breast cancer cells. To this end, SFCM collected from miRZip, miRZip-C, pre-miR and pre-miR-C cells were used to treat MDA-MB-231 cells for 24 h. Subsequently, the migration/invasion of MDA-MB-231 cells were assessed as described above. Figure 4D shows that miRZip-SFCM enhanced the invasion/migration abilities of breast cancer cells (3.35 fold and 2.35 fold, respectively) as compared to control cells that were exposed to miRZip-C-SFCM. However, the invasion/migration of MDA-MB-231 cells were significantly reduced upon incubation with SFCM from pre-miR cells as compared to the corresponding control cells (Figure 4D). This indicates that the expression of miR-146b-5p in breast stromal fibroblasts inhibits the paracrine pro-migration/invasion capabilities of cancer cells. To investigate the possible implication of the pro-migratory/invasive protein kinase AKT in this process, we tested the activation of this protein in miRZip cells as compared to control cells. Figure 4E shows that while miR-146b-5p inhibition did not affect the level of total AKT, the level of the active/phosphorylated form of the protein was strongly increased as compared to control cells. This explains the increase in the invasion/migration and suggests the possible induction of the epithelial-to-mesenchymal transition (EMT) process. To confirm this, we checked the expression levels of epithelial and mesenchymal markers in MDA-MB-231 cells exposed to SFCM from miRZip and miRZip-C cells. Figure 4E shows that SFCM from miR-146–5p-defective fibroblasts decreased the level of E-cadherin, and increased N-cadherin, vimentin and Snail (3 important markers of mesenchymal cells), as compared to their levels in control cells. Together, these results indicate that deficiency of miR-146b-5p in breast stromal fibroblasts triggers EMT in breast cancer cells in a paracrine manner, which demonstrates their active status and their non-cell-autonomous tumor suppressor functions.

### miR-146b-5p level is reduced in breast cancer-associated fibroblasts

**Figure 5:**
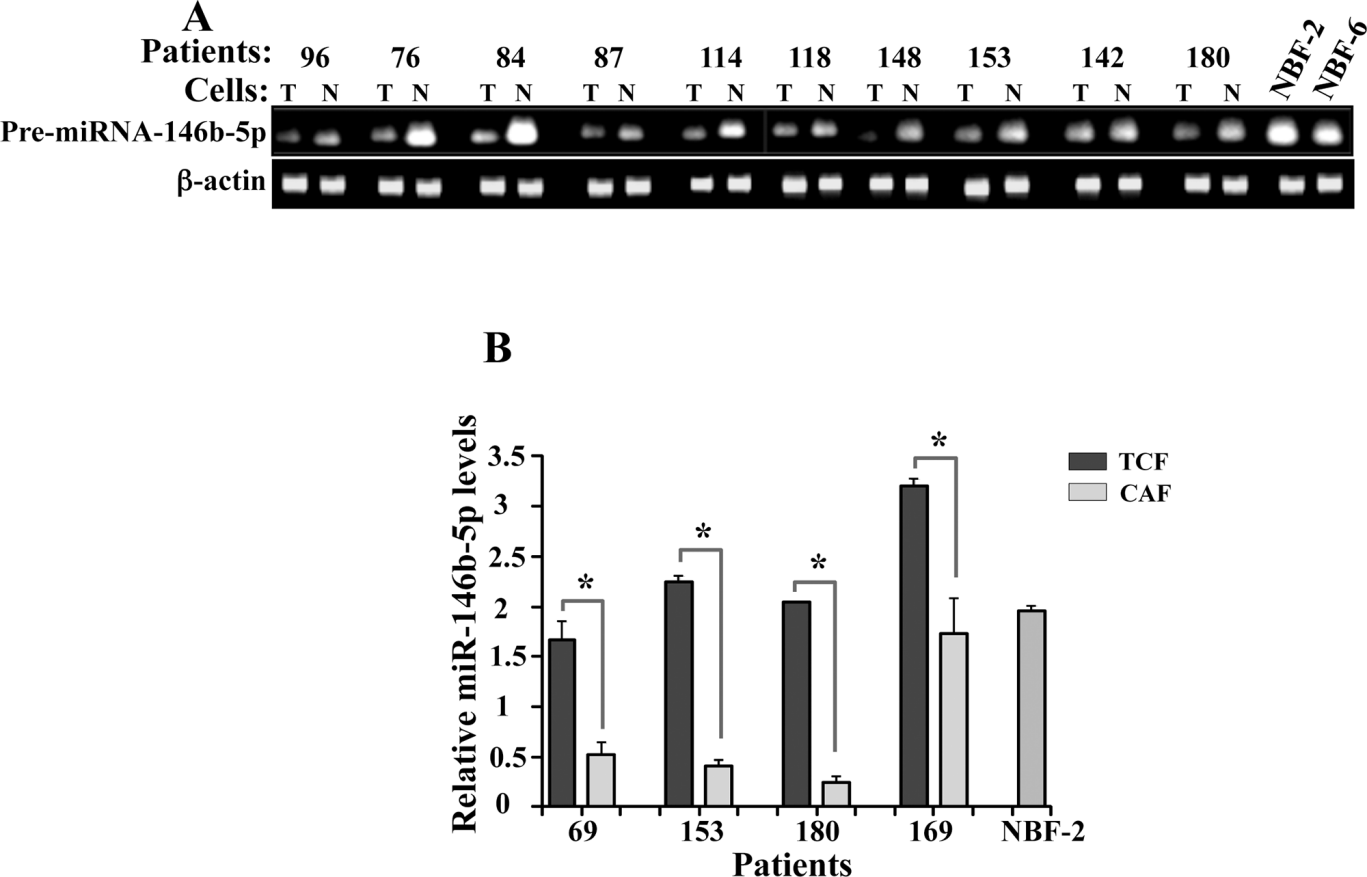
miR-146b-5p is repressed in breast cancer-associated fibroblasts **A.** Total RNA was extracted from the indicated cells and the level of pre-miR-146b-5p was assessed by RT-PCR. The amplified fragments were electrophoresed on ethidium bromide stained agarose gel (N: TCF, T:CAF). **B.** Total RNA was extracted and the level of mature miR-146b-5p was assessed by qRT-PCR. Error bars represent means ± S.D. *p value < 0.031.

Next, we sought to assess the level of miR-146–5p in 10 CAF/TCF pairs from 10 different breast cancer patients and 2 normal breast fibroblast cells (NBF2 and NBF6) developed from normal tissues obtained upon mammoplasty. Total RNA was extracted and specific primers for pre-miR-146–5p as well as β-actin were utilized for amplification by RT-PCR. Figure 5A shows a clear decrease in the pre-miR-146–5p level in all CAFs, as compared to their adjacent TCFs and normal fibroblasts NBF-2 and NBF-6. To further confirm this, we assessed the levels of mature miR-146b-5p in four CAF/TCF pairs and NBF2 by qRT-PCR. Figure 5B shows that the level of the mature form of miR-146–5p is also significantly lower in CAFs as compared to their counterparts TCFs and NBF-2. This indicates that the miR-146b-5p level is reduced in cancer-associated fibroblasts.

### Curcumin induces miR-146b-5p

**Figure 6:**
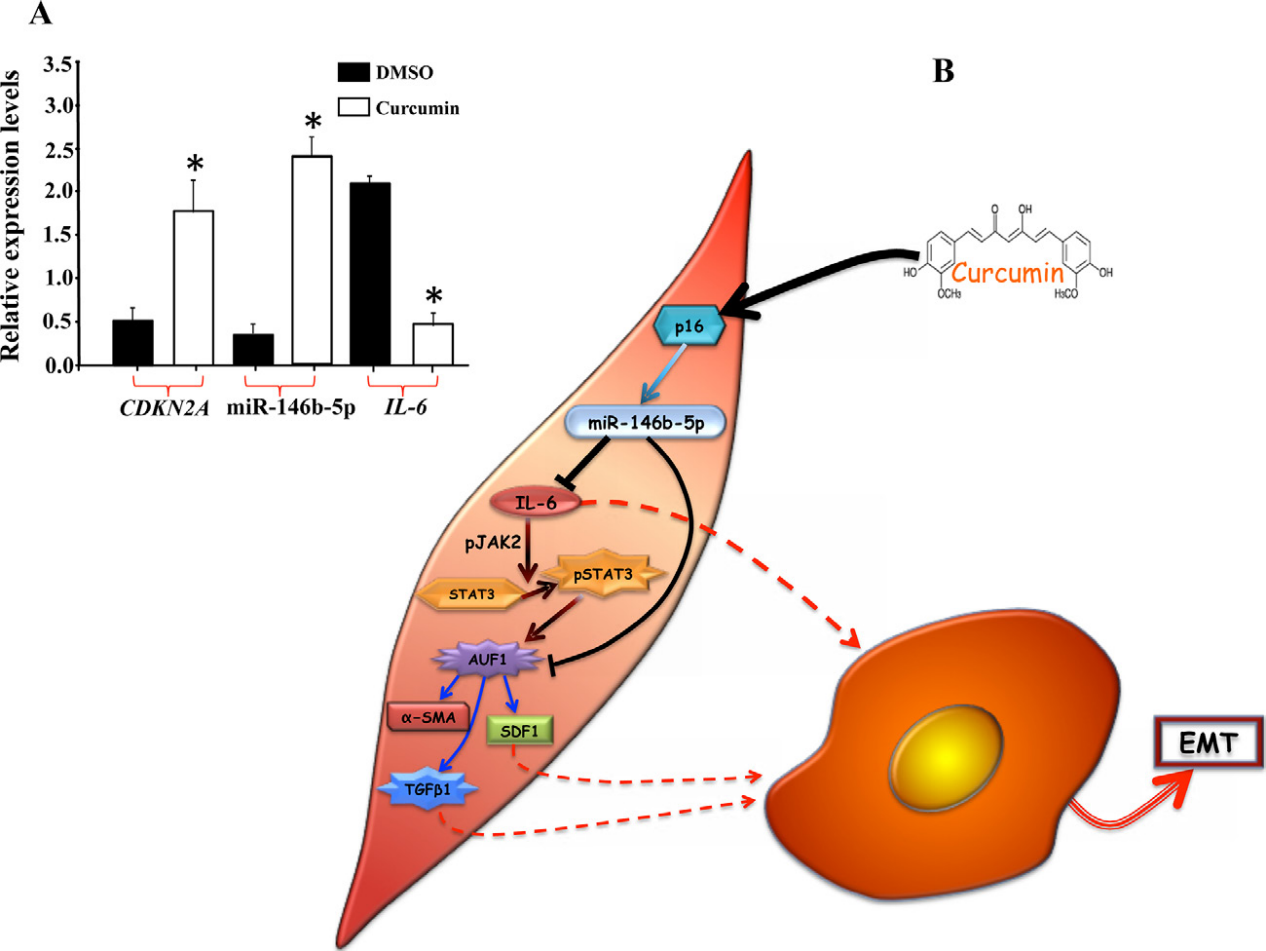
Curcumin induces miR-146b-5p **A.** CAF-64 cells were treated with DMSO or challenged with curcumin (40 μM) for 24 h, and then total RNA was purified and the levels of the indicated RNAs were assessed by qRT-PCR. Error bars represent means ± S.D. *p value < 0.0081. **B.** Schematic representation of the role of miR-146b-5p in p16-dependent negative regulation of IL-6 and the consequent repression of breast stromal fibroblasts, and the effect of curcumin. See text for details.

We have recently shown that curcumin induces p16 in breast stromal fibroblasts [22]. Since p16 promotes miR-146b-5p expression, we sought to investigate the possible curcumin-dependent induction of miR-146b-5p and IL-6 inhibition. To this end, CAF-64 cells expressing low level of p16 were treated with DMSO (control) or challenged with curcumin (40 μM) for 24 h, and then total RNA was purified and the levels of the *CDKN2A* and IL-6 mRNAs as well as mature miR-146b-5p were assessed by qRT-PCR. Figure 6A shows curcumin-dependent induction of *CDKN2A* (4 fold) and miR-146b-5p (5 fold). On the other hand, curcumin suppressed IL-6 (4 fold), as compared to control cells (Figure 6A). This shows curcumin-dependent induction of miR-146b-5p and suppression of IL-6 in active breast stromal fibroblasts.

## DISCUSSION

We have recently shown that p16 is frequently repressed in cancer-associated fibroblasts and when knocked-down leads to the transactivation of breast stromal fibroblasts [3]. This suggests that this tumor suppressor gene plays a major role in maintaining stromal fibroblasts in an inactive state, which by inference, represses carcinogenesis. In the present report, we addressed the molecular mechanism underlying the paracrine signaling of p16-defective cells. We have first shown that p16 is an inhibitor of the expression/secretion of IL-6 in breast stromal fibroblasts. The fact that IL-6 is an important inflammatory cytokine suggests that p16 exerts also anti-inflammatory effects in breast stromal fibroblasts through repressing IL-6. As a case in point, induction of p16 into the synovial tissues suppressed rheumatoid arthritis in animal models. Furthermore, p16 has anti-inflammatory functions in rheumatoid synovial fibroblasts *in vitro* [23, 24]. Recently, it has been shown that p16 suppresses IL-6 expression through IRAK1 degradation in LPS-stimulated macrophages, which are the major source of inflammatory cytokines in inflamed synovial tissues [25].

We have also shown that the increase in the expression/secretion of IL-6 in p16-defective cells is responsible for the paracrine pro-invasive/migratory effects of these cells on breast cancer cells. This effect was strongly reduced when IL-6 was specifically inhibited by an anti-IL-6 inhibitory antibody in medium conditioned with active p16-deficient stromal fibroblasts. This suggests that IL-6 plays a major role in the paracrine pro-carcinogenic effects of p16-deficient cells.

Next, we addressed the molecular mechanism underlying p16-dependent repression of IL-6 and have shown that this control is mediated through miR-146b-5p. We have recently shown that p16 is a positive regulator of miR-146b-5p [16], and the present data indicate that this microRNA inhibits IL-6, whose mRNA contains a specific miR-146b-5p binding site in its 3′UTR. Interestingly, ectopic expression of pre-miR-146b-5p restored the expression of IL-6 to a normal level in p16-defective cells. These results were confirmed by showing that the effect of miR-146b-5p on the expression of IL-6 is mediated through binding to its seeding sequence in the *IL-6* 3′UTR.

miR-146b-5p is a tumor suppressor microRNA, which represses various pro-carcinogenic processes such as migration/invasion, EMT [17–19] and also breast cancer metastasis [20]. In addition, we present here the first indication that miR-146b-5p is also involved in repressing breast stromal fibroblasts. Indeed, while specific inhibition of this miRNA reactivated these cells, ectopic expression of pre-miR146b-5p inhibited the migration/invasion abilities of active breast stromal fibroblasts and suppressed their procarcinogenic effects. This indicates that, in addition to its cell autonomous tumor suppressor functions, miR-146b-5p has also non-cell-autonomous activities through repressing the secretion of IL-6. Similar effect has been shown for miR-26 in ERα-positive breast tumors [26], and miR-31 and miR-214 in ovarian cancer [27]. In addition, the expression of several miRNAs has been shown to be modulated in CAFs as compared to their adjacent normal fibroblasts [28]. This indicates that down-regulation of miR-146b-5p in cancer-associated fibroblasts is an important step during breast carcinogenesis. In fact, we have found that CAFs from 12 breast cancer patients expressed low level of miR-146b-5p as compared to their adjacent counterparts present in histologically normal tissues of the same breast. How miR-146b-5p controls the expression of these myofibroblast markers, namely α-SMA, TGF-β1 and SDF1 ? We have recently shown that miR-146b-5p represses AUF1, which is an activator of these genes and a direct target of active STAT3 [5, 29]. Therefore, miR-146b-5p could indirectly suppresses the expression of α-SMA, TGF-β1 and SDF1 through directly targeting AUF1, or indirectly via IL-6/STAT3 (Figure 6B). The effect on these genes may also explain the paracrine pro-EMT and -metastasis effects of miR-146b-5p (Figure 6B).

These results suggest that increasing the level of miR-146b-5p in active stromal fibroblasts could be of great therapeutic value. To address this question we have shown that curcumin induces p16 and its downstream target miR-146b-5p and suppresses IL-6 (Figure 6A). This further confirms the inhibitory effect of p16 and miR146b-5p on IL-6. It is also plausible that curcumin acts directly on the expression of miR-146b-5p. This possible up-regulation of 2 important tumor suppressors, p16 and miR-146b-5p, and the repression of IL-6, opens new avenues for anti-cancer drug development through targeting stromal fibroblasts and their paracrine effects.

## MATERIALS AND METHODS

### Cells, cell culture and chemicals

Breast fibroblast cells were obtained, characterized and cultured as previously described [30, 31]. Breast tissues were obtained from patients who underwent surgery at the King Faisal Specialist Hospital & Research Center. Signed informed consent was obtained from all the patients under the Research Ethical Committee Project number RAC#2031091. While CAFs derived from tumours, TCFs were developed from histologically normal tissues located at least 2 cm away from tumours (invasive ductal carcinomas). Processing of breast cancer tissues was performed after routine examination by certified anatomical pathologist using hematoxilin and eosin (HE)-stained sections. NBF cells derived from healthy age-matched females who performed breast reduction surgery. In the present experiments NBFs, CAFs and their corresponding TCFs were always cultured simultaneously, in the same conditions and at similar passages (4–8). MDA-MB-231 cells were obtained from ATCC, and were cultured following the instructions of the company.

All supplements were obtained from Sigma (Saint Louis, MO, USA) except for antibiotics and antimycotics solutions, which were obtained from Gibco (Grand Island, NY, USA). Cells were maintained at 37°C in humidified incubator with 5% CO_2_.

### Cellular lysate preparation and immunoblotting

This has been performed as previously described [32]. Antibodies directed against N-cadherin, Vimentin (RV202) and interleukin-6 (IL-6) were purchased from Abcam (Cambridge, MA); Snail (C15D3), E-cadherin (24E10) Akt, phospho-Akt (193H12) from Cell Signaling (Danvers, MA); p16^INK4a^ from BD Biosciences (San Jose, CA) and Glyceraldehydes-3-phosphate dehydrogenase (GAPDH, FL-335) was purchased from Santa Cruz (Santa Cruz, CA).

### RNA purification, RT-PCR and qRT-PCR

Total RNA was purified using the TRI reagent (Sigma) according to the manufacturer's instructions, and was treated with RNase-free DNase before cDNA synthesis using the miScript^®^ II RT Kit (Qiagen, UK) for both miRNAs and mRNAs as previously described [16]. The sequences of the primers are as fellows:

*β-actin*: 5′-CCCAGCACAATGAAGATCAAGATCAT-3′ and 5′-ATCTGCTGGAAGGTGGACAGCGA-3′; *CDKN2A*: 5′-CAACGCACCGAATAGTTACG-3′ and 5′-CAGCTCCTCAGCCAGGTC-3′;

*SDF1*: 5′-GATTGTAGCCCGGCTGAAGA-3′ and 5′-TTCGGTCAATGCACACTTGT-3′;

*α-SMA*: 5′-CCGACCGAATGCAGAAGGA-3′ and 5′-ACAGAGTATTTGCGCTCCGAA-3′;

*TGF-β1*:5′-TGTGTGCTGAAGCCATCGTTG-3′ and 5′-CCGGCTTGTCTGAAAAGGTCA-3′

*IL-6*: 5′-GACAAAGCCAGAGTCCTTCAGAGA-3′ and 5′-CTAGGTTTGCCGAGTAGATCT-3′

*miR-*146b-5p: 5′-CCTGGCACTGAGAACTGAAT-3′ and 5′- GCACCAGAACTGAGTCCACA-3′

### Transfection

*CDKN2A*-shRNA expressed in pRNAT-U6/Neo vector (GenScript Corporation) and the corresponding control plasmid were used to carry out transfection using human dermal fibroblast nucleofector kit (Amaxa Biosystems) following the protocol recommended by the manufacturer.

pLKO.1-miRZip146b-5p and pCDH-pre-miR-146b-5p plasmids were purchased from System Biosciences (SBI, USA) and used to prepare the lentiviral supernatant. Media was removed from the target cells and replaced with lentiviral supernatant and incubated for 24 h.

### Viral infection

Lentivirus based vector bearing *CDKN2A*–ORF (pIRES) as well as it's respective control (Addgene) was used to prepare the lentiviral supernatant from 293FT cells. Lentiviral supernatants were collected 48 h post-transfection, filtered and used for infection. 24 h later, media was replaced with complete media and cells were grown for 3 days.

### Flow cytometry

Cells were harvested and resuspended in 1 ml of PBS before being fixed by drop-wise addition of 3 ml of 100% methanol. Fixed cells were centrifuged, resuspended in 50 ml of RNase (1 mg/ml) and incubated for 30 min at room temperature, followed by addition of 1 ml of 0.1 mg/ml of propidium iodide (PI). Cells were analyzed for DNA content by flow cytometry (Becton Dickinson). The percentage of cells in various cell-cycle phases was determined by using Cell Quest software (Becton Dickinson).

### ELISA assays

Supernatants from 24 hr fibroblast cell cultures were harvested, and ELISA was performed according to the manufacturer's instructions (R&D Systems or RayBiotech for MMP-2). The OD was used at 450-nm on a standard ELISA plate-reader. These experiments were performed in triplicates.

### Chemotaxis and invasion assay

The 24-well BD BioCoat Matrigel Invasion Chambers were used as per the manufacturer guideline (BD Bioscience). 2–4 × 10^5^ cells were added to the upper wells separated by an 8 micron pore size PET membrane with a thin layer of matrigel basement membrane matrix (for invasion) or without (for migration). The membranes were stained with Diff Quick stain (Fisher Scientific) after removing the non-migrated cells from the top of the membrane with Q-tips. After air-drying, the membranes were cut and mounted on slides with oil, and cells that had migrated to the underside of the filter were counted using light microscope (Zeiss Axio Observer) in five randomly selected fields (magnification; 40x). Each assay was performed in triplicate.

### Conditioned media

Cells were cultured in media +/− serum for 24 hr, and then media were collected and centrifuged. The resulting supernatants were used either immediately or were frozen at −80°C until needed.

### Statistical analysis

Statistical analysis was performed by student's *t*-test and *p* values of 0.05 and less were considered as statistically significant.
